# Voluntary exercise and cardiac remodeling in a myocardial infarction model

**DOI:** 10.1515/med-2020-0109

**Published:** 2020-06-11

**Authors:** Hamad Al Shahi, Tomoyasu Kadoguchi, Kazunori Shimada, Kosuke Fukao, Satoshi Matsushita, Tatsuro Aikawa, Shohei Ouchi, Tomoyuki Shiozawa, Shuhei Takahashi, Yayoi Sato-Okabayashi, Koji Akita, Kikuo Isoda, Tetsuro Miyazaki, Hiroyuki Daida

**Affiliations:** Department of Cardiovascular Medicine, Juntendo University Graduate School of Medicine, Tokyo, Japan; Sportology Center, Juntendo University Graduate School of Medicine, Tokyo, Japan; Graduate School of Health and Sports Science, Juntendo University, Chiba, Japan; Department of Cardiovascular Surgery, Juntendo University Graduate School of Medicine, Tokyo, Japan; Faculty of Health Science, Juntendo University, Tokyo, Japan

**Keywords:** voluntary exercise, myocardial infarction, cardiac remodeling, inflammation, myokine

## Abstract

We investigated the effects of voluntary exercise after myocardial infarction (MI) on cardiac function, remodeling, and inflammation. Male C57BL/6J mice were divided into the following four groups: sedentary + sham (Sed-Sh), sedentary + MI (Sed-MI), exercise + sham (Ex-Sh), and exercise + MI (Ex-MI). MI induction was performed by ligation of the left coronary artery. Exercise consisting of voluntary wheel running started after the operation and continued for 4 weeks. The Ex-MI mice had significantly increased cardiac function compared with the Sed-MI mice. The Ex-MI mice showed significantly reduced expression levels of tumor necrosis factor-α, interleukin (IL)-1β, IL-6, and IL-10 in the infarcted area of the left ventricle compared with the Sed-MI mice. In the Ex-MI mice, the expression levels of fibrosis-related genes including collagen I and III were decreased compared to the Sed-MI mice, and the expression levels of IL-1β, IL-6, follistatin-like 1, fibroblast growth factor 21, and mitochondrial function-related genes were significantly elevated in skeletal muscle compared with the Sed mice. The plasma levels of IL-6 were also significantly elevated in the Ex-MI group compared with the Sed-MI groups. These findings suggest that voluntary exercise after MI may improve in cardiac remodeling associated with anti-inflammatory effects in the myocardium and myokine production in the skeletal muscles.

## 1 Introduction

Left ventricular (LV) remodeling leads to chronic heart failure and remains a major source of morbidity and mortality after myocardial infarction (MI). An MI results in instant tissue damage due to myocardial ischemia, followed by biochemical changes that are triggered by reperfusion and pathological remodeling [1]. The loss of myocardial tissue and the consequently increased hemodynamic load on the remaining myocardium induce hypertrophy of the myocytes, myocardial extracellular matrix remodeling, and rearrangement of the myocytes [2]. However, despite the progress in our understanding of the pathophysiological processes of MI and the use of pharmacological interventions in recent decades, post-MI mortality remains high [3].

Inflammatory responses are known to play a major role in the initiation, progression, and destabilization of atherosclerosis [4]. An acute MI triggers an acute inflammatory response – which helps to repair the heart – but excessive inflammatory responses lead to adverse LV remodeling and heart failure [5]. In addition to local inflammation in the myocardium, patients who have experienced an acute MI have been shown to exhibit an amplified systemic inflammatory response, that includes elevations of circulating inflammatory levels of cytokines, chemokines, and cell adhesion molecules, and the activation of various types of immune cells [6,7,8].

Exercise training is a non-pharmacological intervention for the improvement of cardiac function and skeletal muscle function [9], and it is also associated with reductions of the risk of coronary artery disease and heart failure through direct and indirect mechanisms by which exercise contributes an anti-inflammatory effect [10]. We and another research group demonstrated that in atherosclerotic mice, voluntary exercise ameliorated the progression of atherosclerotic lesion formation by exerting anti-inflammatory effects [11,12]. In addition, the benefits of exercise training on skeletal muscle induce mitochondrial adaptations that are characterized mainly by increased mitochondrial biogenesis and the regulation of energy metabolism [13]. These results suggested that skeletal muscle might mediate the anti-inflammatory effects of exercise via secretion of proteins that could counteract the harmful effects of excessive inflammation. We thus conducted this study to assess the effects of voluntary post-MI exercise on cardiac function, remodeling, and inflammation, including myokine production, in a mouse model.

## 2 Methods

### 2.1 Experimental groups

Eight-week-old male C57BL/6J mice were randomly assigned to the following four groups: sham-operated mice (Sh) and mice with MI that were sedentary (Sed-Sh and Sed-MI) and sham-operated mice and mice with MI that were subjected to voluntary exercise training (Ex-Sh and Ex-MI) for 28 days. After the 28-day training period, echocardiography was performed for all mice. The mice were then sacrificed, and the heart and skeletal muscle tissues were excised. Total RNA and protein were isolated from these tissues. All animal experiments were reviewed and approved by the Institutional Animal Care and Use Committee at Juntendo University, Graduate School of Medicine.

### 2.2 Experimental procedures and exercise protocol

Mice were subjected to MI by ligation of the left anterior descending coronary artery (LAD) or to a sham operation without ligation [14]. In brief, the mouse was anesthetized with an intraperitoneal injection of pentobarbital sodium (50 mg/kg) and then intubated and connected to a rodent respirator. The chest cavity was opened via a left thoracotomy to expose the heart, and LAD was visualized by microscopy and permanently ligated with a 7-0 silk suture at the site of its emergence from the left atrium. Complete occlusion of the vessel was confirmed by the presence of myocardial blanching in the perfusion bed. After operation, the sedentary groups were maintained under usual care, whereas the mice in the exercise groups were allowed to exercise immediately by engaging in voluntary wheel running for 28 days as described [11].

### 2.3 Echocardiography

Echocardiographic evaluations were performed at baseline and on day 28 after the operation. The anterior and posterior LV wall thickness, LV end-diastolic diameter (LVEDD), and LV end-systolic diameter (LVESD), the ejection fraction (EF), and the fractional shortening (FS) percentages were measured in mice anesthetized with 1.5% isoflurane. M-mode tracings were obtained with the use of a transthoracic 2D M-mode echocardiographic system (Vevo 770, VisualSonics, Toronto, Canada) [15].

### 2.4 Histology and immunohistochemistry analyses

Heart tissue was fixed in 10% buffered formalin, embedded in paraffin, and cut into 5-µm-thick sections. The sections were stained with Masson’s trichrome to determine the percentage of the infarct size, and the morphology of the infarcted size was identified using ImageJ software (ver. 1.47v; U.S. National Institutes of Health, Bethesda, MD). The percentage of the infarct size was assessed as the total infarct circumference divided by the total LV circumference × 100, as described [16]. Immunohistochemistry staining was performed by the immune-peroxidase method using paraffin-embedded tissue sections. After inhibition of endogenous peroxidase activity, the sections were incubated with primary rat anti-mouse Mac-3 antibody (#550292, BD Bioscience, Franklin Lakes, NJ), or rabbit anti-human CD3 antibody (#20006172F, Dako Autostainer/Autostainer Plus, Dako, Glostrup, Denmark) and then incubated at 4°C overnight with the respective secondary antibodies. Following visualization with 3,3′-diaminobenzidine (#10107863, Sigma–Aldrich, St. Louis, MO) according to the manufacturer’s instructions, the sections were finally counterstained with Mayer’s hematoxylin (Wako, Tokyo). All images were digitized using a microscope (Olympus AX80; Olympus Optical, Tokyo) equipped with a high-resolution camera (Nikon D2X; Nikon, Tokyo).

### 2.5 RNA extraction and quantitative RT-PCR

Total RNA from the heart and skeletal muscle tissue were isolated using the RNeasy Mini Kit (Qiagen, Valencia, CA). We prepared complementary DNA from the total RNA by using reverse transcriptase (Applied Biosystems, Foster City, CA) [17]. Specific mRNAs were amplified using SYBR Premix Ex Taq II (Takara Biotechnology, Shiga, Japan) in an ABI PRISM 7500 thermal cycler (Applied Biosystems). Quantitative real-time polymerase chain reaction (PCR) was performed using a 7500 Real-Time PCR system (Applied Biosystems). The relative amounts of mRNA were calculated by the comparative computed tomography method with glyceraldehyde-3-phosphate dehydrogenase (GAPDH) mRNA as the invariant control.

### 2.6 Immunoblotting

Immunoblotting was performed as described [18]. In brief, skeletal muscle tissue samples were homogenized in 1× cell lysis buffer (Cell Signaling, Danvers, MA), supplemented with 1× complete protease inhibitor cocktail (Roche, Basel, Switzerland), and 1 mmol/L phenylmethylsulfonic fluoride. After sonification and centrifugation at 15 000*g* for 10 min at 4°C, the supernatants were collected. Protein aliquots were taken for a total protein assay (Pierce BCA, Rockford, IL) and the remaining 20 µg of lysate was added onto 4–20% gradient gels (Bio-Rad, Hercules, CA), electrophoretically separated by sodium dodecyl sulfate-polyacrylamide gel using a running buffer, and transferred by electroblotting to a polyvinylidene fluoride membrane (Bio-Rad) using a transfer buffer at 20 V overnight. After the membranes were blocked in Tris-buffered saline buffer with 0.1% Tween-20 (TBST) in 5% non-fat dry milk, they were incubated overnight at 4°C with primary antibodies (dilution 1:1,000) against the peroxisome proliferator-activated receptor gamma coactivator (PGC) 1α (#ab54481, abcam, Cambridge, MA), sirtuin 1 (SIRT1) (#07-131, Merck Millipore, Darmstadt, Germany), and mitochondrial transcription factor A (mtTFA) (#ab131607, abcam). After washing three times in TBST buffer, the membranes were incubated with secondary antibodies conjugated with horseradish peroxidase (dilution 1:5,000; Santa Cruz Biotechnology, Santa Cruz, CA). The membranes were washed again in TBST and incubated with the chemiluminescence detection reagent in the Amersham ECL Western Blotting Analysis System (GE Healthcare, Chalfont St Giles, UK) for enhanced chemiluminescence. Equal loading of protein was verified by immunoblotting with GAPDH (Cell Signaling). The proteins were quantified (band × volume) using a LAS-3000 Imaging System (FujiFilm, Kanagawa, Japan).

### 2.7 Measurement of plasma levels of cytokines

The plasma levels of tumor necrosis factor-alpha (TNF-α), interleukin (IL)-1β, IL-6, and IL-10 were determined using commercial multiplexed fluorescent bead-based immunoassays (Milliplex Map Kit; EMD Millipore, Billerica, MA) and measured by a Luminex 200 xPONENT 3.1 System (EMD Millipore). In the experimental design, the base kit can be used with any combination of the four analyte-specific bead sets for greater flexibility. All the samples were measured in duplicate. The assays were performed according to the manufacturer’s instructions.

### 2.8 Statistical analysis

Data are expressed as the mean and standard error of the mean (SEM) and tested for significance using Student’s *t*-test or a one-way analysis of variance with Tukey’s test for post-hoc analysis. All statistics were carried out using GraphPad Prism® ver. 6.0e (GraphPad Software, San Diego, CA). Results were considered significant when the *p*-value was <0.05.

## 3 Results

### 3.1 Changes in body weight, exercise performance, and survival

At 28 days post-MI, the body weights (BWs) were significantly lower in the exercise groups than in the sedentary groups (Table 1). The values of heart weight (HW) and the HW/BW ratio were significantly higher in the Sed-MI and Ex-MI groups compared with the corresponding sham groups. However, there was no significant difference in HW or the HW/BW ratio between the Sed-MI and Ex-MI groups after MI. The lung weight (LW) was significantly higher in the Sed-MI group compared with the sham and Ex-MI groups. However, the LW/BW ratio was significantly higher in the Sed-MI groups compared with the sham group. Voluntary exercise had no significant effect on the survival rate (Sed-MI: 76%, Ex-MI 68%, *p* = 0.42). The exercise distances per day at 28 days after MI were not significantly different between the Ex-Sh and Ex-MI groups (13 ± 0.3 vs 10 ± 0.2 km/day, respectively; *p* = 0.33). Moreover, the total exercise distance over the 28 days after MI was significantly different between the Ex-Sh and Ex-MI groups (117 ± 5 vs 98 ± 4 km, respectively; *p* < 0.05).

**Table 1:** Characteristics of WT mice after sham or MI operation

	Sedentary		Exercise	
	Sham	MI	Sham	MI
*n*	11	10	10	9
Initial BW, g	23.5 ± 0.3	23.1 ± 0.3	23.8 ± 0.3	23.1 ± 0.3
Final BW, g	27.6 ± 0.5*	27.8 ± 0.5*	25.2 ± 0.5*^†^	25.8 ± 0.4*^†^
HW, mg	112 ± 3	160 ± 7^#^	123 ± 4	151 ± 4^#^
LW, mg	129 ± 3	160 ± 9^#^	134 ± 3	139 ± 4^†^
HW/BW	4.0 ± 0.1	5.7 ± 0.2^#^	4.9 ± 0.1	5.9 ± 0.1^#^
LW/BW	4.7 ± 0.1	5.8 ± 0.3^#^	5.3 ± 0.1	5.4 ± 0.2

^#,†^Results are mean ± SEM. *p < 0.05 vs initial BW, ^#^ *p <* 0.05 vs corresponding sham, ^†^ *p* < 0.05 vs Sed-MI. BW, body weight; HW, heart weight; LW, lung weight; MI, myocardial infarction.

### 3.2 Exercise improved cardiac remodeling after MI

**Figure 1:**
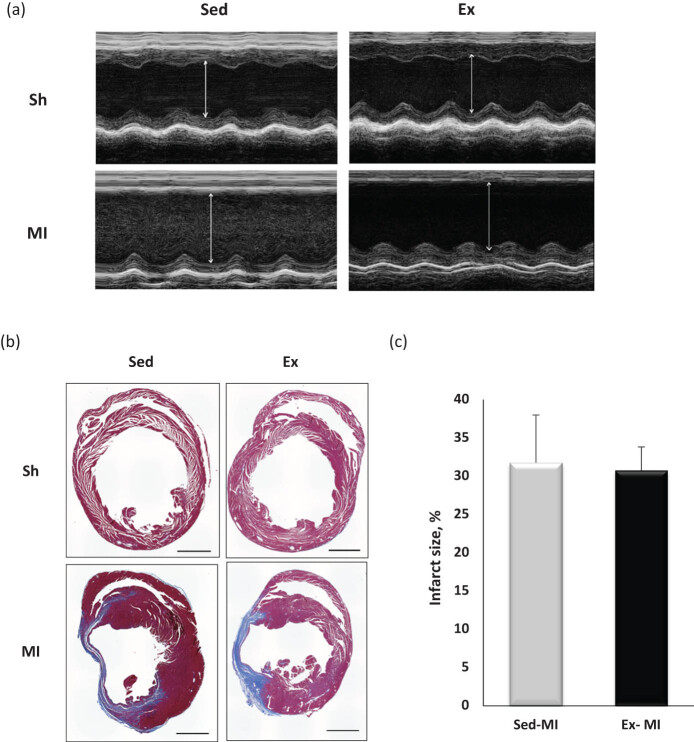
Exercise improved cardiac remodeling after MI. M-mode echocardiographic images obtained from Sed-Sh, Sed-MI, Ex-Sh, and Ex-MI mice at 28 days post-operation (a). Trichrome-stained heart sections (28 days post-MI) (b). Percentage of infarct size in Sed-MI (*n* = 4) and Ex-MI (*n* = 4) (c). Scale bar, 1 mm. Results are given as mean ± SEM. Arrows indicate LV chamber diameter. Ex: exercise, MI: myocardial infarction, Sed: sedentary, Sh: sham.

Heart rates were not significantly different among the four groups (Table 2). The values of LVEDD and LVESD were significantly higher and the EF and FS values were significantly lower at 28 days post-MI in the Sed-MI and Ex-MI groups compared with the corresponding sham groups (Table 2). The values of LVEDD and LVESD were significantly lower in the Ex-MI group compared with the Sed-MI group (*p <* 0.05) (Figure 1a and Table 2). However, there were no significant differences in the EF or FS values between the Ex-MI and Sed-MI groups. The infarct size showed no significant difference between the Sed-MI and Ex-MI groups (Figure 1b and c).

**Table 2:** Echocardiographic measurements

	Baseline				4 weeks			
	Sedentary		Exercise		Sedentary		Exercise	
	Sham	MI	Sham	MI	Sham	MI	Sham	MI
*n*	18	18	17	15	18	18	17	15
HR, bpm	391 ± 9	392 ± 13	373 ± 8	382 ± 8	419 ± 9	412 ± 16	399 ± 6	388 ± 8
LVEDD, mm Hg	3.96 ± 0.04	3.98 ± 0.04	4.12 ± 0.08	3.95 ± 0.04	4.15 ± 0.08	6.32 ± 0.09*^#^	4.27 ± 0.07	5.66 ± 0.05*^#^^†^
LVEDS, mm Hg	2.90 ± 0.04	2.92 ± 0.04	3.07 ± 0.08	2.93 ± 0.04	2.92 ± 0.10	5.72 ± 0.10*^#^	3.15 ± 0.07	5.00 ± 0.06*^#^^†^
EF, %	52.5 ± 1.0	52.2 ± 1.5	50.6 ± 1.5	51.4 ± 0.9	57.0 ± 2.0	20.3 ± 1.0*^#^	52.4 ± 1.5	24.8 ± 1.0*^#^
FS, %	26.6 ± 0.6	26.5 ± 0.9	25.5 ± 0.9	25.9 ± 0.6	29.9 ± 1.5	9.4 ± 0.5*^#^	26.8 ± 1.0	11.6 ± 0.5*^#^

^#,†^Results are mean ± SEM. *p < 0.05 vs baseline, ^#^ *p <* 0.05 vs corresponding sham, ^†^ *p* < 0.05 vs Sed-MI at 28 days post-MI. bpm: beats per minute, EF: ejection fraction, FS: fraction shortening, HR: heart rate, LVEDD: LV end-diastolic diameter, LVESD: LV end-systolic diameter, MI: myocardial infarction.

### 3.3 Exercise suppressed inflammation in the myocardium at 28 days post-MI

**Figure 2:**
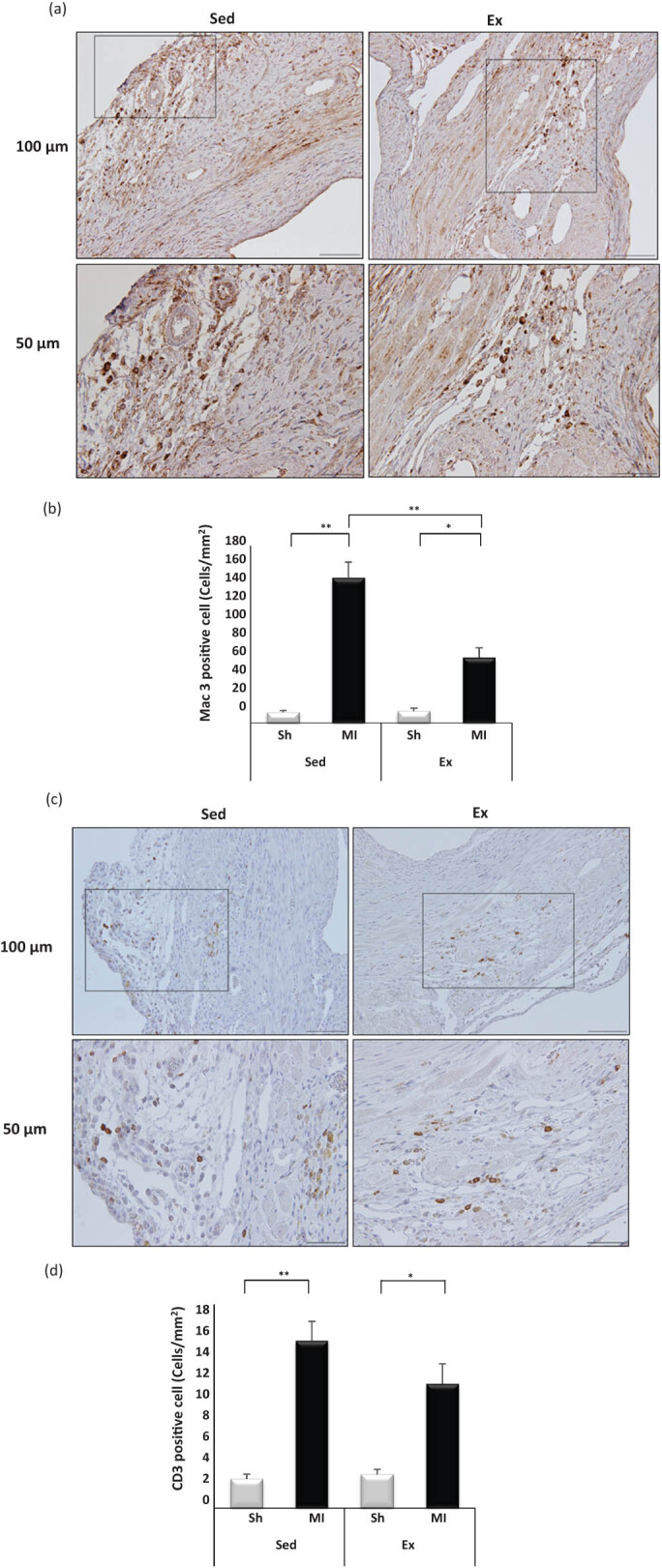
Exercise suppresses inflammation in the myocardium at 28 days post-MI. Immunohistochemistry staining of inflammatory Mac3 cells (macrophage cells) (a). Results of the quantitative analysis of Mac3-positive cells at 28 days post-MI (*n* = 4–5 each) (b). Immunohistochemistry staining of inflammatory CD3 cells (lymphocytes) in the border zone at 28 days post-MI (c). The results of the quantitative analysis of CD3-positive cells at 28 days post-MI (*n* = 4–5 each) (d). Results are given as mean ± SEM. *p < 0.05, **p < 0.001. Ex: exercise, MI: myocardial infarction, Sed: sedentary, Sh: sham.

**Figure 3:**
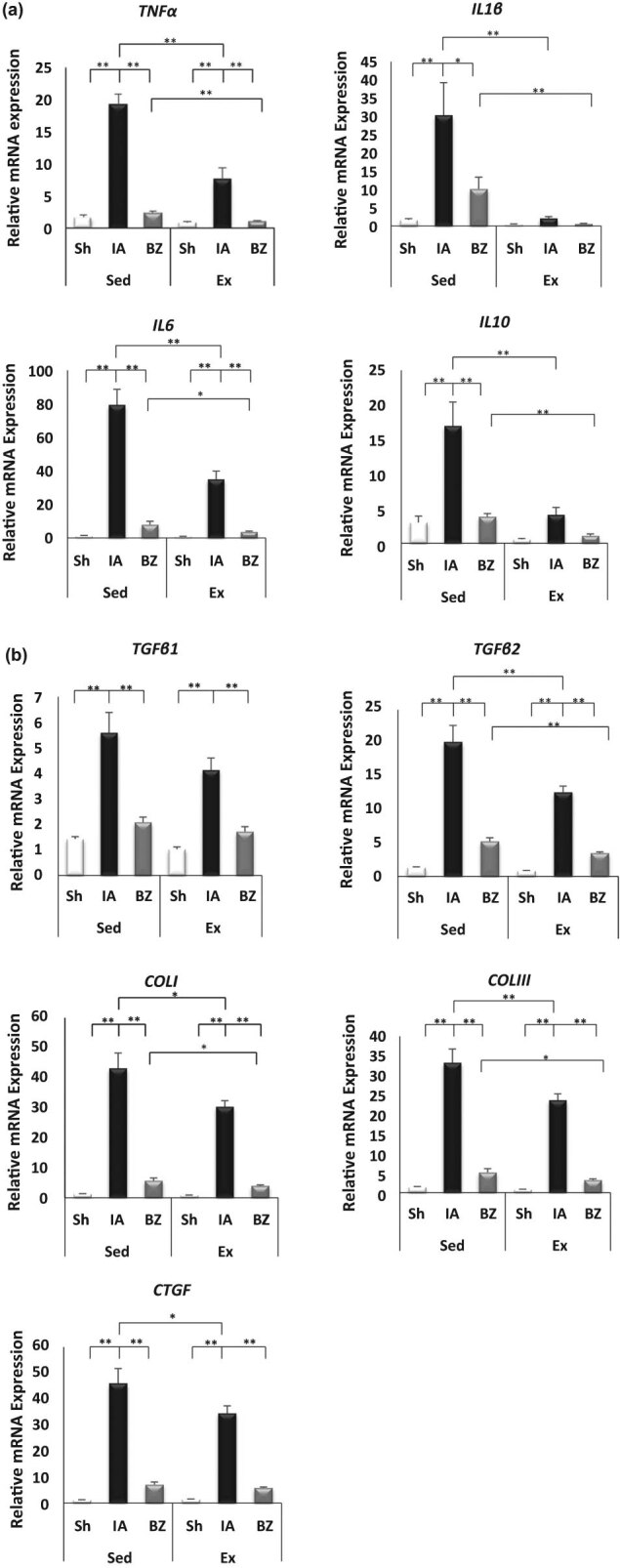
Exercise reduces cardiac fibrosis post-MI. Quantitative analysis of mRNA expression of inflammatory cytokines (*n* = 7–8 each) (a) and fibrosis-related genes in the infarcted area (IA) and border zone (BZ) at 28 days post-MI (*n* = 7–8 each) (b). Results are given as mean ± SEM. *p < 0.05, **p < 0.001. BZ: border zone, COL: collagen, CTGF: connective tissue growth factor, Ex: exercise, IA: infarcted area, IL: interleukin, TGF: transforming growth factor. TNF: tumor necrosis factor, Sed: sedentary, Sh: sham.

Immunohistochemistry staining of Mac-3-positive cells on cardiac tissue sections was carried out 28 days after MI. The infiltration of Mac-3 cells in the border zone of the LV of the Ex-MI mice was significantly decreased compared with the Sed-MI group (66.7 ± 9.8% vs 147.4 ± 15.0%, respectively, *p* < 0.001) (Figure 2a and b). However, the staining of CD3-positive cells in the border zone of the LV at 28 days post-MI showed no significant difference between the Ex-MI and Sed-MI groups (11 ± 1.7% vs 14.8 ± 1.7%, respectively, *p =* 0.17) (Figure 2c and d). The mRNA expression levels of TNF-α, IL-1β, IL-6, IL-10, and IL-27 of the infarcted area of the LV and the border zone of the LV in the Ex-MI group were significantly decreased compared with those of Sed-MI group (*p <* 0.001, Figure 3a).

### 3.4 Exercise reduced cardiac fibrosis after MI

In the infarcted area, the mRNA expression levels of various fibrosis-related genes including transforming growth factor (TGF) β1, TGFβ2, collagen (COL) I, COLIII, and connective tissue growth factor (CTGF) were significantly increased in the Sed-MI and Ex-MI groups compared with the Sham groups (*p <* 0.001). Compared with the Sed-MI group, the Ex-MI group showed significant reductions of the expression of TGFβ2, COLI, COLIII, and CTGF in the infarcted area (*p* < 0.05) and significant reductions in the expression of TGFβ2, COLI, and COLIII in the border zone (*p* < 0.05). TGFβ1 and CTGF showed no significant differences between the Sed-MI and Ex-MI groups in the border zone of the LV (Figure 3b).

### 3.5 Exercise promoted mitochondrial function and myokine expression in skeletal muscle

**Figure 4:**
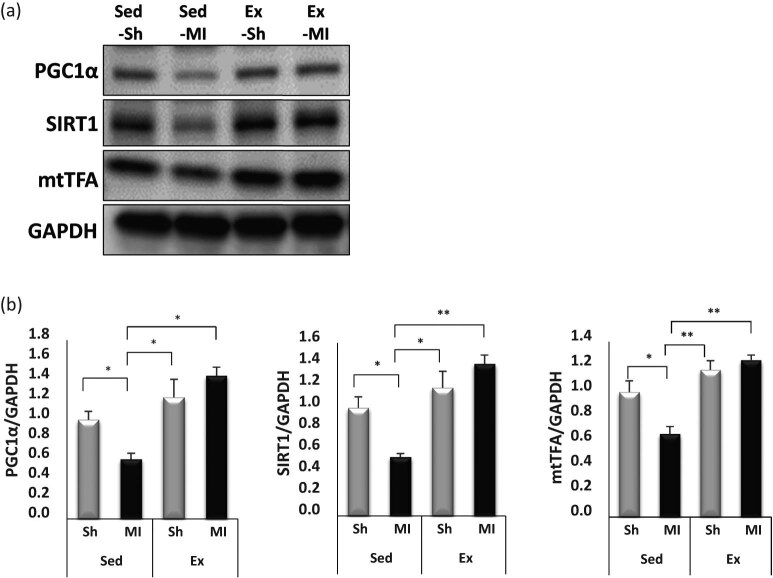
Exercise promoted mitochondrial function in mouse skeletal muscle. Representative immunoblotting bands (a) and results of the quantitative analyses for PGC1α, SIRT1, and mtTFA protein expressions in the gastrocnemius skeletal muscle at 28 days post-MI (*n* = 5 each) (b). Results are given as mean ± SEM. *p < 0.05, **p < 0.001. Ex: exercise, MI: myocardial infarction, mtTFA: mitochondrial transcription factor A, PGC: peroxisome proliferator-activated receptor gamma coactivator, Sed: sedentary, Sh: sham, SIRT: sirtuin.

**Figure 5:**
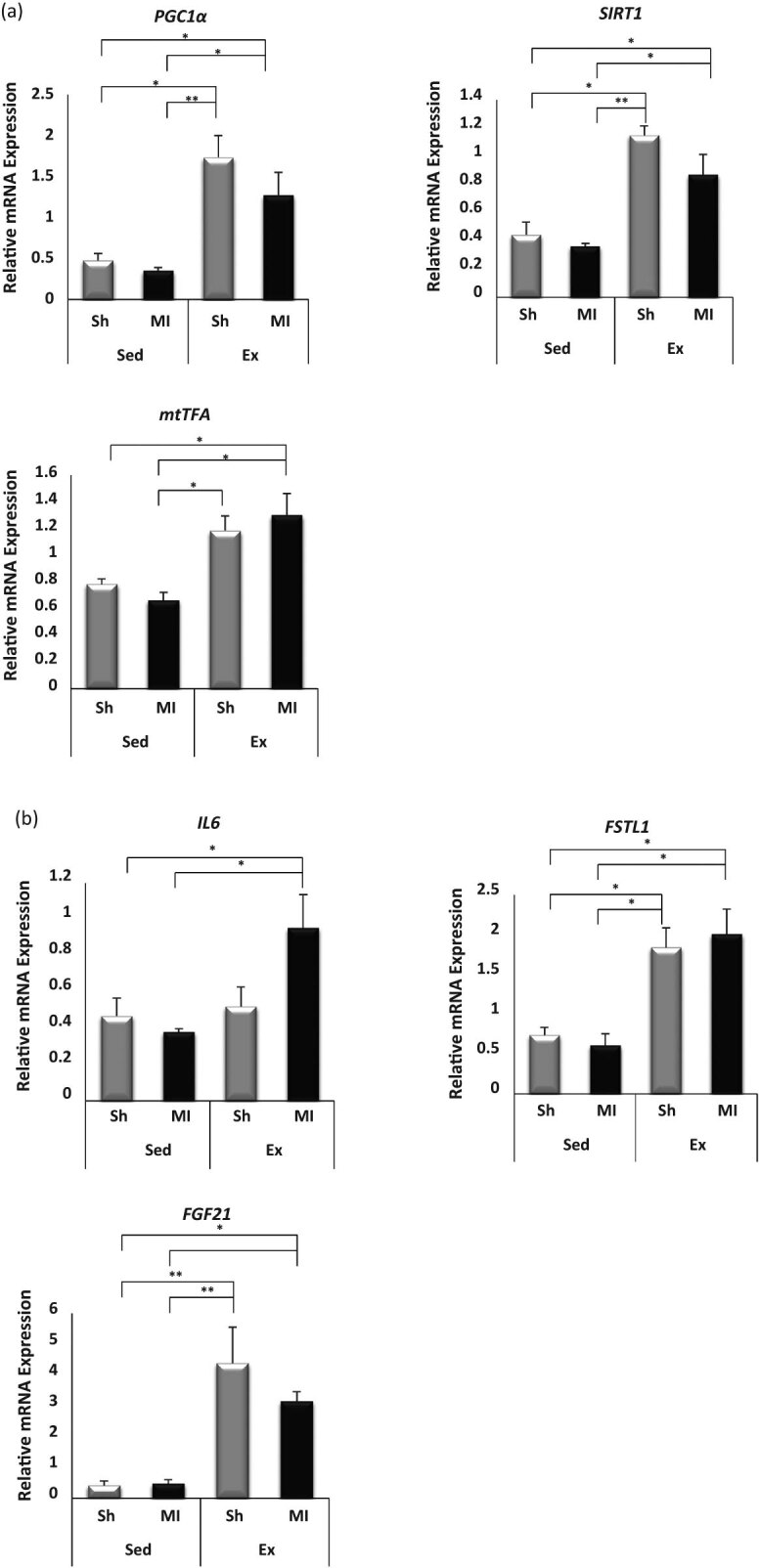
Exercise promoted mitochondrial function and myokine expression in mouse skeletal muscle. Quantitative analysis of mRNA expression of PGC1α, SIRT1, and mtTFA (a), and IL-6, FSTL1, and FGF21 in the gastrocnemius skeletal muscle at 28 days post-MI (*n* = 7–8 each) (b). Results are presented as mean ± SEM. *p < 0.05, **p < 0.001. Ex: exercise, FGF: fibroblast growth factor. FSTL: follistatin-like, IL: interleukin, MI: myocardial infarction, mtTFA: mitochondrial transcription factor A, PGC: peroxisome proliferator-activated receptor gamma coactivator, Sed: sedentary, Sh: sham, SIRT: sirtuin.

To determine whether voluntary exercise provided a stimulus to the expression of various cytokines and exercise-related genes, such as PGC1α, SIRT1, and mtTFA, we performed immunoblotting and RT-PCR analysis (Figures 4a, b and 5a). The voluntary exercise produced a shift toward a greater expression of PGC1α, SIRT1, and mtTFA in the Ex-Sh and Ex-MI groups compared with the Sed-MI group as revealed by immunoblotting (*p* < 0.001). We also observed a significant increase in the mRNA expressions of PGC1α, SIRT1, and mtTFA in the Ex-Sh and Ex-MI groups compared with the Sed-Sh and Sed-MI groups (*p* < 0.05) (Figure 5a).

We also investigated the mRNA expressions in skeletal muscle of myokines, i.e., IL-6, follistatin-like (FSTL) 1, and fibroblast growth factor (FGF) 21 and their potential post-MI roles (Figure 5b). Interestingly, dramatic increases of FSTL1 and FGF21 mRNA expression were observed in the Ex-Sh and Ex-MI mice compared to the Sed-Sh and Sed-MI mice (*p <* 0.05). The IL-6 mRNA expression demonstrated a significant increase only in the Ex-MI mice compared with the Sed-Sh and Sed-MI mice (*p <* 0.05).

### 3.6 Exercise-induced higher IL-6 plasma levels after MI

**Figure 6:**
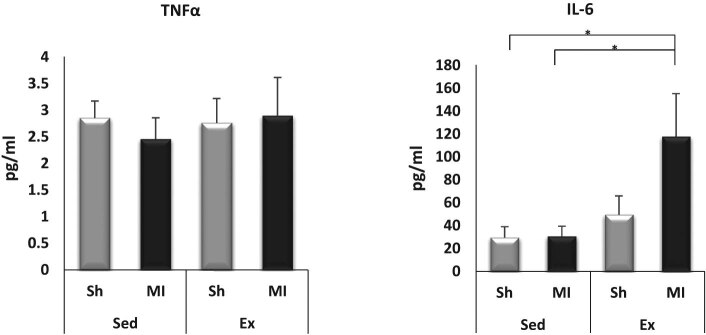
Exercise-induced higher IL-6 plasma levels post-MI. Plasma levels of IL-6 and TNF-α at 28 days post-MI (*n* = 5–7 each). Results are given as mean ± SEM. *p < 0.05, **p < 0.001. Ex: exercise, IL: interleukin, MI: myocardial infarction, Sed: sedentary, Sh: sham, SIRT: sirtuin, TNF: tumor necrosis factor.

The plasma level of IL-6 in the Ex-MI group was significantly increased compared with those of the Sed-Sh and Sed-MI groups (*p <* 0.05, Figure 6). The plasma level of TNF-α showed no significant differences between the groups.

## 4 Discussion

This study results in a mouse model demonstrated that voluntary exercise attenuated cardiac remodeling, modulated inflammatory responses, and induced an improvement in mitochondrial function and an increase in myokine expression in the skeletal muscle after MI.

Other animal studies showed that exercise can promote a protective reaction against irreversible tissue damage produced by ischemic injury in the myocardium [19,20]. Moreover, exercise training may attenuate post-MI remodeling independent of the preconditioning effect. Other investigations revealed that swimming training had no effect on mortality but reduced the infarct size and attenuated LV remodeling in an MI rat model [19,20]. The effects of exercise training after MI on cardiac remodeling and function thus remain incompletely understood.

We observed herein that MI in mice resulted in significant LV remodeling and dysfunction after 28 days, as characterized by increases in LVEDD and LVESD values and decreases in EF and FS values, resulting in pulmonary congestion. However, the exercise training attenuated the LVEDD and LVESD at 28 days post-MI. In addition, the incidence of cardiac hypertrophy in the mice at 28 days post-MI, as indicated by increases in the HW and the HW/BW ratio, was also apparent when compared with the Sham group. These results suggest that voluntary wheel running might be useful for the prevention of cardiac function and remodeling in animal post-MI models.

Myocardial infarction triggers an intense inflammatory reaction that is essential for the healing of the LV infarcted area. Myocardial infarction and reperfusion injury have been associated with the activation of pro-inflammatory cytokines such as TNF-α, IL-1β, and IL-6, and this activation promotes leukocyte activation and extravasation into the LV infarcted area [21,22]. Exercise training was shown to have beneficial effects on the inflammatory response in the heart through the attenuation of LV remodeling [19,23]. In this study, inflammatory cytokines (TNF-α, IL-1β, IL-6, IL-10, and IL-27) were attenuated in the mice that exercised, suggesting that the phase of inflammatory reaction is an important signaling mechanism that contributes to the LV remodeling processes. The pro-inflammatory environment in the early stages of infarct healing promotes matrix degradation and phagocytic clearance, and the repair of the infarcted tissue is dependent on the signaling pathways that mediate the inflammatory responses. In this study, the myocardial expressions of fibrosis-related genes (TGFβ1, TGFβ2, COLI, COLIII, and CTGF) were significantly upregulated post-MI. However, the expressions of TGFβ2, COLI, COLIII, and CTGF in the mice that exercised were downregulated compared with the sedentary group post-MI.

Mitochondrial biogenesis is a complex process that requires the coordinated synthesis and assembly of ∼1,500 proteins encoded by both the nuclear and mitochondrial genomes [24]. PGC1α is known to coactivate multiple mitochondrial transcription factors, leading to the upregulation of fatty acid oxidation, in part through increased PGC1α protein stability induced by the protein deacetylase SIRT1 and in part through mtTFA activation, a key component in the transcription of multiple oxidative genes [25]. In this study, the gene expression and protein content of PGC1α, SIRT1, and mtTFA were significantly decreased in the skeletal muscles Sed-MI group, compared with the Sed-Sh group, whereas they were significantly increased in the exercised MI groups. This suggests that voluntary exercise enhanced the mitochondrial content and oxidative capacity in skeletal muscle.

The role of skeletal muscle in protecting the heart after MI has been studied in animal models [26]. Since exercise has muscle ischemia-like effects through hypoxia, exercise may exert cardiac protective action through a mechanism similar to ischemia [27]. Skeletal muscle, upon contraction, stimulates the production and release of cytokines (which in the muscles are also called myokines), which can influence metabolism and modify further myokines production in the tissues and organs [28]. Some myokines can induce an anti-inflammatory response after exercise training. For example, during exercise, IL-6 is the first detectable myokine released into the blood from the contracting skeletal muscle, and this release induces a subsequent increase in the production of the IL-1 receptor antagonist and IL-10 by the blood mononuclear cells, thereby exerting an anti-inflammatory effect [28]. In this study, the gene expression and plasma circulating levels of IL-6 in Ex-MI mice were significantly elevated by exercise, suggesting that IL-6 may have a beneficial anti-inflammatory effect. Interestingly, in the mice that exercised, the gene expressions of FSTL1 and FGF21 were also upregulated compared with the sedentary mice group. These results are comparable with those of other studies [29,30]. Collectively, our findings suggest that multiple signaling mechanisms may participate in the myokine-mediated cardioprotective role in MI, a possibility that deserves further investigation.

The plasma circulating levels of IL-10 in both the present sham groups were significantly elevated compared with the MI mice. Low expression levels of IL-10 in serum samples have been associated with an increased risk of cardiovascular events, and high IL-10 expression levels have been associated with a decreased risk [31]. Conflicting results have been published [32], and although some studies have found that IL-10 levels were significantly elevated after exercise, further investigations are needed to clarify the relationship between IL-10 levels and exercise.

This study has some limitations. The results were obtained with male mice only and therefore cannot be directly translated to female mice. We were also unable to obtain Milliplex assay data for all the cytokines.

## 5 Conclusion

The results of this study revealed that in the murine model, voluntary exercise after MI ameliorated cardiac remodeling and the inflammatory response and induced an improvement of mitochondrial function and myokine expression in skeletal muscle. These findings suggest that voluntary exercise after MI improves cardiac remodeling via inflammatory modulation. Further research is needed to confirm the beneficial effects of exercise training after MI and to determine how these effects may translate into clinical benefits in human.
